# Targeting Immune Regulatory Networks to Counteract Immune Suppression in Cancer

**DOI:** 10.3390/vaccines4040038

**Published:** 2016-11-04

**Authors:** Chiara Camisaschi, Viviana Vallacchi, Elisabetta Vergani, Marcella Tazzari, Simona Ferro, Alessandra Tuccitto, Olga Kuchuk, Eriomina Shahaj, Roberta Sulsenti, Chiara Castelli, Monica Rodolfo, Licia Rivoltini, Veronica Huber

**Affiliations:** 1 Unit of Immunotherapy of Human Tumors, Fondazione IRCCS Istituto Nazionale dei Tumori, 20133 Milan, Italy; chiara.camisaschi@istitutotumori.mi.it (C.C.); viviana.vallacchi@istitutotumori.mi.it (V.V.); elisabetta.vergani@istitutotumori.mi.it (E.V.); simona.ferro@istitutotumori.mi.it (S.F.); alessandra.tuccitto@istitutotumori.mi.it (A.T.); eriomina.shahaj@istitutotumori.mi.it (E.S.); roberta.sulsenti@istitutotumori.mi.it (R.S.); chiara.castelli@istitutotumori.mi.it (C.C.); monica.rodolfo@istitutotumori.mi.it (M.R.); licia.rivoltini@istitutotumori.mi.it (L.R.); 2 Mount Sinai Liver Cancer Program, Icahn School of Medicine at Mount Sinai, New York, NY 10029, USA; olga.kuchuk@mssm.edu

**Keywords:** immune suppression, cancer, therapy, myeloid-derived suppressor cells, regulatory T cells, extracellular vesicles, tumor acidity

## Abstract

The onset of cancer is unavoidably accompanied by suppression of antitumor immunity. This occurs through mechanisms ranging from the progressive accumulation of regulatory immune cells associated with chronic immune stimulation and inflammation, to the expression of immunosuppressive molecules. Some of them are being successfully exploited as therapeutic targets, with impressive clinical results achieved in patients, as in the case of immune checkpoint inhibitors. To limit immune attack, tumor cells exploit specific pathways to render the tumor microenvironment hostile for antitumor effector cells. Local acidification might, in fact, anergize activated T cells and facilitate the accumulation of immune suppressive cells. Moreover, the release of extracellular vesicles by tumor cells can condition distant immune sites contributing to the onset of systemic immune suppression. Understanding which mechanisms may be prevalent in specific cancers or disease stages, and identifying possible strategies to counterbalance would majorly contribute to improving clinical efficacy of cancer immunotherapy. Here, we intend to highlight these mechanisms, how they could be targeted and the tools that might be available in the near future to achieve this goal.

## 1. Introduction 

Immune suppression is an area of great interest in cancer immunotherapy. Although it has been acknowledged that tumor cells spontaneously induce T cell activation through the expression of specific antigens, and that these responses are likely blunted in vivo by immune escape mechanisms, an impressive tumor control that can be achieved by neutralizing immunosuppressive mechanisms (such as in the case of immune checkpoint inhibitors, ICI) could not be foreseen even by the most optimistic scientists [1]. As a matter of fact, the true revolution that is presently occurring in cancer therapy thanks to the broad clinical efficacy of antagonist antibodies to CTLA-4, PD-1 and PD-L1 is actually unraveling the potency that antitumor immune responses can reach if appropriately unleashed [2]. The worldwide research is thus rightly focused on further exploiting the ICI strategy to improve its therapeutic potential. This could be achieved by combining ICI with standard oncologic treatments, or targeting additional immune checkpoints regulating antitumor immune responses. Nonetheless, preclinical evidence accumulated over the last two decades clearly underlines that different types of cancers can utilize a wide range of pathways to deceive our defenses, developing a hostile microenvironment where, under the pressure of a sort of “Darwinian selection”, only cancer cells and tumor-educated stromal cells can survive and proliferate [3,4,5]. This pressure spreads early to immunologically relevant organs (including lymph nodes and bone marrow) to create permissive systemic conditions by molding the whole immune system in its multiple components [6,7,8]. 

**Figure 1:**
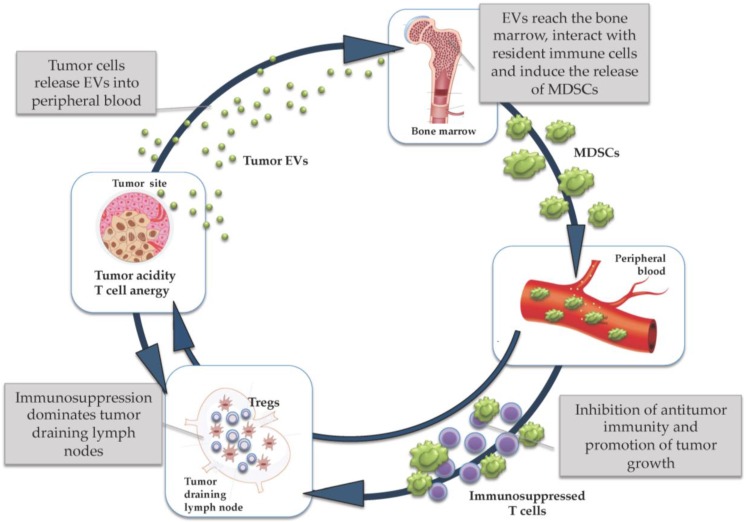
The immunosuppressive cycle.

Each of the steps composing this “immunosuppressive cycle” (Figure 1), if appropriately elucidated by in-depth studies, holds promise to offer novel therapeutic targets to improve immunomodulation in cancer patients. Dissecting some of these steps in melanoma patients has been the major goal of our unit, particularly focusing on pathways involving myeloid-derived suppressor cells (MDSCs), regulatory T cells (Tregs), tumor acidity and the role of tumor extracellular vesicles (EVs). Here, we will discuss which strategies could be applied to interfere with these mechanisms of immunosuppression, in order to improve the clinical efficacy of cancer immunotherapy.

## 2. Counteracting MDSCs, a Major Local and Systemic Obstacle to Immune-Mediated Tumor Control

MDSCs, a heterogeneous mixture of myeloid cells, play a crucial role in both local and systemic immune suppression. At tumor sites, MDSCs provide a microenvironment favoring tumor cell survival and metastasis. In fact, in addition to exerting immune suppression, they possess strong angiogenic functions and directly promote tissue remodeling. Human MDSCs include a monocytic population (M-MDSCs), CD11b^+^CD14^+^HLA-DR^low/−^CD15^−^, and a polymorphonuclear population (PMN-MDSCs), CD14^−^CD11b^+^CD15^+^(or CD66b^+^), formerly called granulocytic-MDSCs [9]. 

Several independent studies demonstrated that the different subsets of MDSCs accumulate in the blood and at the tumor sites of cancer patients [10]. While the accumulation of MDSCs is a phenomenon occurring across tumors of different histologies, it is nevertheless true that different tumors might preferentially accrue a given MDSC subset, and thus the phenotypic features of MDSCs could somehow be shaped by the tumor histotype [11]. In different types of cancer including melanoma [12], bladder [13], and hepatocellular carcinoma [14], the enhanced frequency of circulating M-MDSCs is associated with the clinical stage of the disease and has been shown to correlate with decreased progression-free survival, as demonstrated for late stage melanoma [15]. The level of circulating MDSCs is a negative independent prognostic factor [16] and, moreover, high frequency of MDSCs limits the patients’ response to different immunological and non-immunological treatments [17,18]. Most importantly, immunological monitoring of cancer patients treated with ICI consistently supports the role of MDSCs as major resistance pathway and potential predictive factor of non-response [19,20,21].

Given the strong and multifaceted pro-tumor activity of MDSCs, their association with clinical features and their crucial ability to hamper the patients’ response to therapy, the targeting of these cells represents a promising approach to improve tumor treatment. In this regard, strategies are mainly focused on reduction of MDSC levels in the blood by blocking their expansion from hematopoietic precursors, promotion of MDSC differentiation, and inhibition of their suppressive functions. It is worth noting that blocking MDSC recruitment through chemokine receptor blockade has been recently identified as a promising new strategy in preclinical and clinical studies [22,23]. In this view, the majority of the approaches stems from the findings about the molecular mechanisms of biology, development, recruitment and effector functions of these suppressive cells. In fact, many MDSC functions depend on signaling cascades mediated by different classes of tyrosine kinases [24]. Sunitinib, a drug targeting a wide range of kinases, including those mediating platelet-derived growth factor receptor (PDGFR), vascular endothelial growth factor receptor 1 and 3 (VEGFR1 and VEGFR3), fms like tyrosine kinase 3 (FTL3), stem cell factor (SCF), and macrophage colony-stimulating factor (M-CSF) signaling, is a first line treatment for metastatic renal cell cancer. In addition to its anti-angiogenic functions, sunitinib is endowed with a potent immunomodulating activity, and its administration strongly decreases the level of circulating MDSCs in renal cell cancer patients [25]. Additional data now suggest that other tyrosine kinase blockers, such as dasatinib and sorafenib, are also very efficient in inhibiting MDSC induction [26]. We confirm the immune functions of sunitinib in soft tissue sarcoma patients, where we observed a strong downregulation of both Tregs and M-MDSCs in the blood of patients under sunitinib treatment. We also assessed a correlation between downregulation of M-MDSCs and the duration of response to treatment. Conversely, disease progression was associated to a rebound in the frequency of M-MDSCs. Importantly, these sunitinib resistant M-MDSCs displayed alternative STAT3 activation [27]. Indeed, STAT3 plays a crucial role as signal transducer both in MDSC suppressive activity and proliferation. Inhibition of STAT3 signaling by STAT3-targeted siRNA or treatment with Stattic, a STAT3-specific inhibitor, abrogated the arginase dependent suppressive function of M-MDSCs in head and neck squamous cell carcinoma patients [28]. Additionally, STAT3 is a valuable target for abolishing the suppressive activity of PMN-MDSCs, as shown in prostate cancer patients. STAT3 inhibition might also promote the differentiation of MDSCs and induce immature myeloid cells toward a more mature, HLA-DR positive status, as shown for the circulating M-MDSCs of advanced lung carcinoma patients treated with Cucurbitacin B (CuB), a selective inhibitor of the JAK2/STAT3 pathway [29,30]. Several new compounds able to block STAT3 activation are currently being tested in ongoing phase II clinical trials, and it is likely that some of their efficacy might be ascribed to their interference with the activities of MDSCs. Nevertheless, it must be mentioned that MDSCs present at the tumor site might indeed show resistance toward STAT3 inhibition. In fact, it has been shown very recently that due to the hypoxic conditions prevailing in the tumor microenvironment, MDSCs downregulate STAT3 activity mediated by CD45 tyrosine phosphatase upregulation [31]. 

In addition to drugs targeting STAT3, other compounds have been shown to block the effector functions of patients’ derived MDSCs in vitro and in preclinical models. Celecoxib (Cyclooxygenase-2 inhibitor) suppresses the activation of melanoma derived M-MDSCs and increases the frequency of cytotoxic T lymphocytes (CTLs) [32]. In animal models, phosphodiesterase-5 (PDE-5) inhibitors (sildenafil, tadalafil) reduced MDSC functions by downregulating the expression of arginase 1, inducible nitric oxide synthase (iNOS), and IL-4α in MDSCs [33]. In tadalafil-treated head and neck squamous cell carcinoma patients, the reduction of circulating MDSCs was paralleled by an increase in antitumor immunity, as measured by delayed type hypersensitivity and immune response to head and neck squamous cell carcinoma tumor lysate in vitro [34]. Upregulation of reactive oxygen species (ROS) is one of the main mechanisms of MDSC activity, and their intracellular formation is strongly inhibited by synthetic triterpenoids, such as C-28 methyl ester of 2-cyano-3,12-dioxooleana-1,9,-dien-28-oic acid. This compound, administered to pancreatic cancer patients, limited the MDSC-mediated immune suppression by inducing the upregulation of several antioxidant genes including NAD(P)H, quinone oxidoreductase 1 (NQO1), thioredoxin, catalase, superoxide dismutase, and heme oxygenase, while it did not affect the frequency of circulating MDSCs [35]. The next generation of triterpenoid omaveloxolone (RTA-408) is currently under investigation in phase I/IIb clinical trials for melanoma patients in combination with the checkpoint inhibitors ipilimumab and nivolumab (NCT02259231). Therapeutic interventions resulting in limiting the peripheral frequency of MDSCs includes the treatment with bortezomib in multiple myeloma patients [36], tadalafil for head and neck squamous cell carcinoma [34] and vemurafenib for melanoma [37]. Additional studies, mainly conducted in the pre-clinical murine setting, indicate a role of gemcitabine, 5-fluoruracil and doxorubicin in reducing the frequency, trafficking and recruitment of MDSCs at tumor sites [38,39,40]. However, the effects of these drugs in a human setting still need to be carefully explored since, to date, conflicting data have been reported. This might also be due to the complexity of MDSC identification and studies, due to their different phenotypes, thus making direct comparisons difficult. 

Other strategies of MDSC targeting include attempts in inducing their differentiation. For this purpose, All-trans retinoic acid (ATRA) and 25-hydroxyvitamin D(3) have been administered to renal cell cancer and head and neck squamous cell carcinoma patients in pilot studies. Results showed an effective induction of the differentiation of myeloid cells. Of note, 25-hydroxyvitamin D(3) reduced the number of immune suppressive CD34^+^ cells, while increasing HLA-DR^+^ cells and IL-12 and IFN-γ plasma levels. This testifies to an increased activation of DCs and T cells in head and neck cancer patients [41,42]. In renal cell cancer patients, ATRA significantly improved the myeloid/lymphoid dendritic cell ratio and the ability of patients' mononuclear cells to stimulate allogeneic T cells [43]. Finally, as discussed more extensively below, local acidity and hypoxia have recently been shown to foster the suppressive and pro-inflammatory functions of MDSCs as well as their mobilization [44]. Thus, reversing these conditions would indirectly target MDSCs and contribute to limiting their detrimental immune suppressive effects. 

In conclusion, MDSCs represent a diversified population of cells for both phenotype and functions. They play a crucial role in immunosuppressive circuits, and, to date, several strategies are available for limiting their frequency, survival and functions (Table 1). Many of these approaches still need to be validated in the clinical setting. Importantly, new approaches are now designed based on newly discovered features of these cells, such as their dependency from a local hypoxia and acidosis.

## 3. Overcoming the Restraining Activity of Tregs on Adaptive Immunity

Tregs are fundamental for the maintenance of peripheral tolerance and to prevent autoimmunity. However, they can infiltrate human tumors and limit antitumor immunity and immunotherapy effects [45]. Several approaches aimed at directly blocking or depleting intratumoral Tregs exhibited clinical benefits in different tumor mouse models [46]. The use of drugs such as daclizumab, a monoclonal antibody (mAb) against CD25, and denileukin diftitox, an IL-2 diphtheria toxin fusion protein, has been recently translated into the clinic. However, the effects of these drugs and the clinical outcome of patients seem to be highly related to the type of treated malignancy, with severe side effects reported for metastatic melanoma [47,48]. However, since in these studies, different administration schedules have been used, and the actual ability of these molecules in depleting Tregs has not been firmly demonstrated, it is not feasible to evaluate the real impact of Tregs in these tumors. Moreover, a temporal or an incomplete depletion of Tregs can give rise to new Treg rebounds, due to conversion of T cell precursors [49], a mechanism hardly demonstrable ex vivo in patients. With these premises, Treg depletion appears to be rather difficult or even impossible, and thus the attention should be focused on inhibition of their suppressive activity. 

Some traditional anti-cancer drugs have been shown to interfere with Treg activity. The most common chemotherapeutic agent studied for this purpose is cyclophosphamide (CTX). The rationale for investigating the effects of CTX on Tregs is that Tregs residing in the tumor microenvironment are highly proliferating. Actually, only the metronomic administration of CTX seems to possess immunomodulatory effects on Tregs in patients with advanced cancers [50,51]. However, results remain contradictory, potentially due to different treatment schedules and methods applied for Treg detection. Moreover, data obtained in our laboratory suggest that the evaluation of the tumor microenvironment is fundamental to assess the effects mediated by CTX on Tregs. In fact, we have demonstrated that, in the peripheral blood mononuclear cells (PBMCs) of stage II–III melanoma patients enrolled in a phase II randomized trial of vaccination with modified tumor peptides, only a limited and transient modulation of Tregs could be detected. On the other hand, we could observe a significantly lower frequency of Tregs in lymph nodes, surgically removed after two rounds of CTX, as compared with those of untreated patients. Moreover, the lymph node microenvironment also resulted in being less immunosuppressed, as shown by reduced levels of TGFβ and IL-10 cytokine production [52]. Inhibitory effects on Tregs have been also reported after the administration of targeted therapies, such as the tyrosine kinase inhibitors sorafenib, sunitinib and imatinib [53,54,55]. We have recently observed that in soft tissue sarcoma patients, the administration of sunitinib induced a strong reduction in the frequency of circulating Tregs and that this reduction strictly depends on the administration of the drug. In fact, a strong rebound in Tregs occurred immediately when patients suspended the sunitinib treatment [27]. 

Enthusiasm is increasing for the effect of the FDA approved ICI, anti-CTLA-4 and -PD-1 mAbs, which are improving the overall survival (OS) of patients with several types of advanced malignancies, even if the majority of patients (about 70%) still fail to respond to these treatments. Immune checkpoints (IC) can be expressed not only by effector T cells but also by Tregs. While the role of ICI on effector T cells is largely documented [56], their action on Tregs is not totally understood. Indeed, the study of IC in Tregs and the effects of ICI on Tregs in cancer patients are important emerging areas of investigation. However, the real challenge remains the identification of a marker that can univocally identify Tregs. In fact, Tregs are endowed with high plasticity and modulate their phenotype and functions in response to the local milieu [57]. Markers like CTLA-4 and GITR, together with CD25 and Foxp3, have been considered lineage markers, constitutively expressed by Tregs. Other molecules, including LAG-3, TIGIT, PD-1, PD-L1, OX40 and TIM-3 are expressed only by subtypes of Tregs. Furthermore, each of these markers can be expressed by other T cell subtypes, including activated effector T cells, preventing them from being used as exclusive markers to target Tregs. Thus, the use of ICI could have a dual effect on both effector T cells and Tregs. Both CTLA-4 and GITR are constitutively expressed by Tregs. After the administration of ipilimumab, an anti-CTLA-4 mAb, a reduction of Tregs has been demonstrated [58]. However, more than a depletion of Tregs, the positive effects of the anti-CTLA-4 mAbs seem to be due to the interference with the suppressive activity of Tregs. In fact, it has been demonstrated that anti-CTLA-4 mAb restores the activity of memory T cells in metastatic melanoma patients. In particular, these cells become resistant to Treg-mediated suppression [59]. Moreover, Romano and colleagues recently suggested that anti-CTLA-4 therapy may induce Treg lysis in vivo with an antibody-dependent cell-mediated cytotoxicity (ADCC) mechanism mediated by nonclassical monocytes (CD16^++^CD14^+^), thus with an indirect mechanism [60]. Evidence for a direct mechanism of action came from Wang and colleagues. They found that PD-1 blockade induces the down-modulation of Foxp3 expression in Tregs and promotes the generation of melanoma antigen-specific CTLs, suggesting that PD-1 is implicated in the regulation of Treg function [61]. Positive antitumor results have been also demonstrated using anti-GITR and anti-OX40 mAbs in mouse models [62]. In contrast with CTLA-4, GITR and OX40, which both belong to the TNF receptor superfamily, co-stimulatory molecules are expressed both by effector T cells and Tregs. Thus, the administration of these molecules, currently entered into the clinic, could have a dual action: co-stimulatory for the effector T cells and inhibitory for the Tregs. 

A key goal would be to unravel mechanisms or pathways selectively used by Tregs confined to the tumor microenvironment but not in their activity of “normal” immune control. While investigating Treg subpopulations specifically enriched in cancer patients, we identified a discrete CD4^+^ Treg subset characterized by the expression of LAG-3 [63]. These highly suppressive LAG-3 Tregs were expanded in PBMCs of melanoma patients and selectively enriched in lymphocytes of tumor-invaded lymph nodes and in lymphocytes infiltrating visceral metastasis, indicating a possible role of the tumor microenvironment for their recruitment or differentiation at the tumor site. LAG-3 is one of the inhibitory immune checkpoints that has been demonstrated to play a functional role in antitumor immune responses. In ovarian cancer, NY-ESO-1 specific CD8^+^ T cells expressing both LAG-3 and PD-1 are endowed with impaired effector function. Dual blockade of LAG-3 and PD-1 during the T cell priming phase can restore the antitumor function of NY-ESO-1 specific CD8^+^ T cells [64], but the effects on Tregs have not been reported in this work. Only more recently, the clear implication of Tregs has been demonstrated by Goding and colleagues in a mouse model of melanoma. These authors showed that anti-PD-L1 Ab or the depletion of Tregs alone failed to reverse tumor recurrence. Interestingly, both the combination of anti-PD-L1 mAb with Treg depletion and the combination of anti-PD-L1 with anti-LAG-3 mAbs gave rise to the same positive results, indicating that the addition of anti-LAG-3 mAb can substitute for Treg depletion [65]. These encouraging results opened to the possibility of the use of anti-LAG-3/PD-1 combinatory therapy also in humans, with a phase I clinical trial currently recruiting patients (NCT01968109). 

In summary, to counteract immune suppression mediated by Tregs, the main focus should be placed on contrasting their suppressive activity, rather than on elimination of these cells. This could be achieved through the administration of ICI targeting molecules not only expressed by effector T cells but also by Tregs like PD-1, or in combination with novel drugs targeting Treg molecules such as LAG-3 (Table 2). 

## 4. EVs as Conveyors of Immune Suppression, Therapeutic Vehicle and Therapeutic Target in Cancer

Production and release of extracellular vesicles (EVs), devoted to intercellular communication, is a feature shared by normal as well as transformed cells. Since their discovery more than three decades ago, EV studies have dissected their multifaceted roles, mainly depending on their cellular origin. The accumulating evidence prompted possibilities of intervention for the employment of these vesicles as therapeutic targets, agents or vehicles and biomarkers in diseases not limited to cancer. EVs appear to be suitable natural carriers for drugs or other therapeutic agents. At the same time, these vesicles can be “shaped” by insertion of molecules for different purposes, such as targeting. The large family of EVs includes various members, including exosomes, microvesicles, ectosomes, apoptotic bodies and large oncosomes [66]. Each vesicle type differs from the others in dimension, origin, protein expression or particular content. However, they also share a variety of characteristics, adding up to the complexity of their identification [67]. Among EVs, exosomes released by tumor cells have gained major attention over the years for their tumor-promoting role [68]. Our unit has been devoted to the study of these vesicles since 2001, contributing to the discovery of major effects mediated by tumor exosomes in limiting immune responses by the induction of T cell apoptosis and the skewing of monocyte differentiation into MDSCs [69,70,71]. We were able to identify analogue suppressive cells in peripheral blood of advanced stage melanoma patients, corroborating our in vitro findings. Thereby, we discovered one of the hallmark populations involved in immune suppression in patients affected by cancer [12]. Exosomes derived from cancer cells have been shown to be involved also in the conversion or generation of another major immune cell population involved in suppressive circuits, the Tregs [72]. Since EVs can be found in body fluids like blood (plasma is the fluid mostly studied by EV researchers), urine, milk and saliva, these naturally occurring vesicles have lately found their way into the clinics as biomarkers of disease, including cancer [73,74,75,76]. 

The exploitation of EVs, especially exosomes, as acellular vehicles for the stimulation of antitumor immune responses in vivo has gained major interest starting from more than a decade ago. The attraction of exosomes as an immunotherapeutic tool is based on studies showing that exosomes produced by immune cells (including DCs and B lymphocytes) display functional major histocompatibility complex (MHC) class I and II as well as co-stimulatory and cell adhesion molecules. Zitvogel et al. demonstrated for the first time in 1998 that exosomes secreted by bone marrow-derived DCs, which were challenged with tumor-derived peptides, activated CTLs and caused the eradication of established tumors in mice [77]. Administration of exosomes to cancer patients has been performed in phase I and II clinical trials, primarily using exosomes derived from dendritic cells (Dexosomes) and IFNγ-Dexosomes in melanoma and lung cancer patients [78]. Other clinical approaches included the administration of tumor exosomes to boost immune responses, due to the cargo of tumor antigens carried by these nanovesicular structures and their uptake by antigen presenting cells [79]. An important issue to exploit tumor exosomes for therapeutic purposes will be to optimize their targeting properties, as discussed by Kunigelis and Graner. In fact, these authors emphasize the necessity to target administered tumor exosomes to antigen presenting cells, avoiding thereby their detrimental effects on T and natural killer (NK) cells and promoting at the same time tumor antigen presentation and antitumor immune response [80]. 

The possibility of loading specific molecules, drugs and genetic material into and onto EVs has attracted major interest for their exploitation as therapeutic shuttles to tumor sites [81]. Our unit has recently tested the efficacy of antitumor activity of exosomes originating from tumor necrosis factor-related apoptosis-inducing ligand (TRAIL)-transduced cells and carrying membrane TRAIL in preclinical studies [82]. Apart from targeting tumor cells, the proapoptotic activity of TRAIL exosomes could be exploited to limit immune suppression by eliminating TRAIL death receptor-expressing MDSCs [83]. EVs endowed with endogenous killing properties like NK cell derived exosomes could also contribute to overcoming immune suppression. These vesicles are produced spontaneously by NK cells, can be found as circulating vesicles in healthy donors and cancer patients, and are involved in immune surveillance. Natural killer cell-derived exosomes were found to express activatory molecules on their surface, indicating that they may be involved in interfering with immune suppressive circuits [84]. In addition, exosomes derived from bovine milk appear as versatile and cost-effective tools to carry drugs to tumor sites, advantaged by the presence of tumor targeting ligands, as shown in preclinical models [85]. Successful preclinical approaches have been achieved with plant exosomes, due to their natural anticancer properties. Grapefruit and citrus-limon derived exosomes have been tested in vitro as well as in vivo with interesting results in complete absence of toxicity [86,87]. Loading of exosomes with the curcumin, a plant with anticancer and anti-inflammatory effects, represents another strategy pursued by different groups [88]. The effects mediated by plant-derived exosomes, which can be also loaded with genetic material to target tumor cells such as specific miRNAs, display their anti-inflammatory potential also on leukocytes, contributing thereby to retaining immune suppression. 

Among the multitude of approaches currently under evaluation, the capture of tumor exosomes aimed at dampening their detrimental effects on the immune system appears as an interesting strategy. Selective elimination of exosomes from the body implicates their recognition, this being a particularly difficult task. In this view, Gobbo and coworkers developed an interesting strategy based on the expression of hsp70 primarily by cancer exosomes, as detected in the plasma of cancer patients. With the help of an hsp70 binding aptamer, these authors were able to demonstrate the inhibition of MDSC activation mediated by hsp70 carrying exosomes binding to TLR2 expressed by MDSCs [89].

The discovery of genetic material inside of the extracellular vesicles led to enormous development in EV research. In fact, this opened up new functional aspects of these vesicles, offering new possibilities for therapeutic targeting approaches. The loading of EVs with selected miRNAs appears to be a major strategy with potential translation into the clinics. Currently, the miRNA selection is mainly aimed at inhibiting tumor growth. To date, few approaches actually target cells involved in immune suppression. In fact, the targeting of therapeutic vesicles to immune cells might represent a new challenge. However, to overcome immune suppression in cancer, we might learn from the induction of immune suppression in autoimmunity diseases, such as contact allergies. Here, the targeting of immune cells is the principal objective, as demonstrated by a recent study showing the delivery of a chosen inhibitory miRNA to target effector T cells in an antigen-specific manner by a surface coating of antibody light chains [90]. Additionally, the selective elimination of exosomes from the circulation by extracorporeal hemofiltration has been proposed as an alternative therapeutic strategy [91]. Indeed, we and other groups have demonstrated that cancer exosomes not only contribute to immune dysfunctions but can also interfere with therapeutic antibodies, such as the anti-HER2 trastuzumab or anti-CD20 rituximab, administered to patients affected by B cell lymphoma [92,93]. 

It becomes evident that EVs, depending on their cells of origin and their content and composition, could be utilized to overcome immune suppression in cancer patients mainly by contrasting their immune suppressive effects through elimination, if originating from tumor and immune suppressive cells, or by exploiting their potential as shuttles to distant sites to target immune suppressive cells like Tregs and MDSCs to revert immune suppressive status. In this regard, due to their bioavailability, the exploitation of EVs, especially exosomes, appears as an attractive alternative for drug or molecule delivery purposes. Moreover, EVs can be targeted to reach not only the tumor site, but also immune suppressive cells present in the tumor microenvironment. It could be envisaged that, in the near future, exosome therapeutics might be composed of different “ingredients” aimed at docking specific targets and modulating the suppressive milieu.

## 5. Tumor Acidity as a Novel Target for Immunomodulation

The mechanisms linking chronic inflammation and cancer are not fully clarified, but certainly involve multiple interactions between cancer and stroma immune cells. These complex networks are hypothesized to be driven by a variety of soluble factors including proinflammatory cytokines/chemokines, directly inhibiting tumor immunity and/or polarizing resident, or recruited myeloid cells toward immunosuppressive or protumorigenic phenotypes [94]. However, a concept recently emerging as a novel hallmark of cancer is the ability to reprogram energy metabolism and consequently create altered metabolic conditions in the tumor microenvironment [95]. As a consequence of this overactive metabolism, cancer cells are burdened with toxic byproducts, including protons and lactate, which are then expelled through pH regulators (including H^+^-ATPases, Na^+^-H^+^ antiporters, and H^+^-linked monocarboxylate transporters), leading to decreased pH in the tumor microenvironment [96]. 

Acidity can profoundly mold immune responses, impairing T cell proliferation and function, but also favoring the differentiation of proinflammatory stromal cells [3]. Recent work from our unit showed that tumor-specific T cells lose their effector properties and enter an anergy state when exposed to low pH, facing a functional paralysis that highly resembles that mediated by inhibitory immune checkpoints, and that can be reverted in vitro by pH buffering [97]. In a murine melanoma model, the administration of the anti-acid drug esomeprazole (contrasting tumor acidity through the inhibition of vATPase) can increase CD8^+^ T cell infiltrate in tumor lesions and significantly potentiate the therapeutic efficacy of a cancer vaccine or adoptive immunotherapy [97], supporting the improved onset of antitumor immune responses in buffered pH conditions [98]. In addition to a direct inhibitory effect on effector T cells, acidity can impair adaptive immunity by promoting the accumulation of immunosuppressive cells. For instance, MDSCs have been shown to acquire the majority of their immunosuppressive and protumor activities when entering tumor hypoxic/acidic environments, including the expression of immune checkpoints such as PD-L1 [99,100]. Furthermore, low pH promotes secretion by cancer cells of immunosuppressive cytokines or chemokines enhancing the recruitment of Tregs and MDSCs [101]. On the basis of this evidence, the biochemical consequences of cancer-related dysmetabolism may represent an upstream and highly efficient pathway of tumor immune escape at the microenvironment level. In this view, tumor acidity may represent a novel target to modulate immunosuppression by interfering with cancer metabolism. The abovementioned esomeprazole and the whole family of proton pump inhibitors (PPI) hold promise of being potent tools for immunomodulation and appealing drugs in cancer treatment. Indeed, in addition to reverting T cell anergy through pH buffering, proton pump inhibitors interfere with cancer metabolism, inducing tumor cell apoptosis and significantly potentiating sensitivity to chemotherapy both at preclinical and clinical levels [102,103,104].

In addition to vATPases, several pH regulators such as carbonic anhydrases (CA) IX and XII expressed in cancers have been mostly investigated for their role in tumor cell proliferation and survival [105]. Nevertheless, data are emerging about their role in conditioning adaptive or innate immunity; CAIX, for instance, has been recently identified as being involved in promoting MDSC mobilization and in the establishment of pre-metastatic niches through a G-CSF-mediated pathway [106]. In a non-tumor setting, CA has been found to promote mast cell-mediated immunity, which is reported to promote immunosuppression and sustain tumor growth in several cancer models [107]. Small molecules, antibodies and off-target drugs targeting CA regulators are currently at various stages of clinical development, giving the possibility to modulate tumor-associated acidic microenvironments and study the implications of different components of tumor immune response readily transferable into clinical settings in the near future. Of note is the evidence that treatment of metastatic renal cell carcinoma with autologous T lymphocytes genetically retargeted against CAIX mediates clinical responses points to the further possibility of targeting pH regulators and tumor associated acidity through antigen-specific immunotherapy [108]. 

An easy albeit non-specific approach to counteract tumor acidity is represented by bicarbonate monotherapy, which has been shown to impair growth of some cancers in preclinical models, and to contrast tumor development in prostate cancer-prone mice [109]. More recently, combining bicarbonate administration with anti-CTLA-4 or anti-PD-1 mAbs, or adoptive T cell transfer, has been reported to improve antitumor responses in multiple murine models, further emphasizing the need to overcome acidity-related immunosuppression to optimize immune-mediated tumor control [110].

## 6. Conclusions

Enormous progress has been achieved over the last few years in the field of cancer immunotherapy, thanks to the identification of the right tools to unleash adaptive and innate immunity and its potency in controlling tumor growth and progression. Nevertheless, the pathways of immune suppression addressed so far, mostly related to the upregulation of immune checkpoints, involve only a subset of the complex cross-talk networking progressively established by cancer cells with host environments during disease development. Although many of these mechanisms might be common features of tumor cells, as clearly demonstrated by broad efficacy of PD-1/PD-L1 blockade across different cancer types, some of them could instead be linked to the histological nature of the transformed tissue, the surrounding organ, and the biophysical/biochemical properties of the microenvironment. Furthermore, the reaction that the immune system establishes in response to diverse patterns of immune suppression might be finely tuned in different settings, resulting in multiple molecular pathways that could display histotype- and even patient-specificity. Understanding these intricate and dynamic scenarios more in depth, and, most importantly, developing proper means for clinical intervention will surely pave the way to an effective immune modulation, for a comprehensive way to cure cancer by counteracting immune suppression.

## Figures and Tables

**Table 1:** Inhibitors of myeloid-derived suppressor cells (MDSCs).

Therapeutic Agent	Specification	Histology (Mice and Humans)	Direct or Indirect Effects on MDSCs	References
PF-04136309	CCR2 inhibitor	Pancreatic cancer	Blocking of MDSC recruitment	[22,23]
Sunitinib	Tyrosine kinase inhibitor	Metastatic renal cancer, soft tissue sarcoma	Decrease of circulating MDSCs Downregulation of M-MDSCs	[25,27]
Stattic	Small molecule inhibitor of pSTAT3	Head and neck squamous cell carcinoma	Targets arginase dependent suppressive function of M-MDSCs	[28]
Cucurbitacin B (CuB)	JAK2/STAT3 pathway inhibitor	Advanced lung carcinoma	Promotes MDSC differentiation	[29]
Celecoxib	Cyclooxygenase-2 inhibitor	Melanoma cells	Suppression of melanoma derived M-MDSC activation	[32]
Sildenafil, tadalafil	Phosphodiesterase-5 (PDE5) inhibitor	Murine colon, breast cancer, fibrosarcoma	Reduction of MDSC functions	[33]
Tadalafil	Phosphodiesterase-5 (PDE5) inhibitor	Head and neck squamous cell carcinoma	Reduction of circulating MDSCs	[34]
CDDO-Me; bardoxolone methyl	Triterpenoid	Pancreatic cancer; murine colon, lung, thymus cancer	Abrogation of the suppressive effect of MDSCs	[35]
Bortezomib	Proteasome inhibitor	Multiple myeloma	Reduction of circulating MDSCs	[36]
Omaveloxolone (RTA-408)	Triterpenoid	Melanoma	Abrogation of the suppressive effect of MDSCs	NCT02259231
Vemurafenib	B-rapidly accelerated fibrosarcoma (BRAF) inhibitor	Melanoma	Inhibition of M-MDSC generation	[37]
Gemcitabine, 5-fluoruracil, doxorubicin	Chemotherapeutic agent	Murine thymoma, breast cancer	Reduction of MDSC frequency, trafficking and recruitment	[38,39,40]
25-hydroxyvitamin D(3)	Vitamin	Head and neck cancer, Murine lung carcinoma	Differentiation of MDSCs, reduction of immune suppressive CD34(+) cells	[41,42]
All-trans retinoic acid (ATRA)	Vitamin	Renal cell cancer	Decrease of MDSCs by induction of differentiation	[43]

**Table 2:** Inhibitors of regulatory T cells (Tregs).

Therapeutic Agent	Specification	Histology (Mice and Humans)	Direct or Indirect Effects on Tregs	Reference
Daclizumab	Monoclonal antibody against CD25	Advanced melanoma	Treg depletion from the peripheral circulation	[47]
Ontak (Denileukin Diftitox)	IL-2 diphteria toxin fusion protein	Advanced melanoma	No evident Treg elimination	[48]
Cyclophosphamide (low dose/metronomic administration)	Chemotherapeutic agent	Advanced cancers Advanced-stage breast cancer	Profound and selective reduction of circulating Tregs, associated with a suppression of their inhibitory functions Only transient Treg reduction, but it induces stable tumor-specific T cell responses, which correlate with improved clinical outcome	[50,51]
Sorafenib	Tyrosine kinase inhibitor	Renal cell carcinoma	Reduction of the percentage of tumor-infiltrating Tregs	[54]
Sunitinib	Tyrosine kinase inhibitor	Metastatic renal cancer Soft tissue sarcoma	Decrease of the number of peripheral blood and intratumoral Tregs Reduction of the frequency of circulating Tregs	[27,53]
Imatinib	Tyrosine kinase inhibitor	Murine leukemia and lymphoma	Decrease of Treg frequency and impairment of immunosuppressive function	[55]
Anti-CTLA-4 mAb	Immune checkpoint inhibitor	Advanced melanoma	T cell becomes resistant to Treg-mediated suppression It can engage ex vivo nonclassical monocytes resulting in ADCC-mediated lysis of Tregs	[59,60]
Anti-PD-1 mAb	Immune-checkpoint inhibitor	Advanced melanoma	Down-regulation of intracellular FoxP3 expression	[61]
Anti-PD-L1 and anti-LAG-3 mAbs	Immune checkpoint inhibitors	Murine Melanoma	Simultaneous blockade of PD-L1 and LAG-3 in vivo overcomes the necessity to deplete tumor-specific Tregs	[65]
Anti-OX40 and anti-GITR mAbs	Co-stimulatory molecules, TNF receptor superfamily	Murine GVHD	Abrogation of Treg suppression of GVHD	[62]

## Abbreviations

The following abbreviations are used in this manuscript:

CA	carbonic anhydrases
CTLs	cytotoxic T lymphocytes
EVs	extracellular vesicles
ICI	immune checkpoint inhibitors
MDSCs	myeloid-derived suppressor cells
Tregs	regulatory T cells
